# Pathological staging of chorioamnionitis contributes to complications in preterm infants

**DOI:** 10.1186/s13052-020-00895-4

**Published:** 2020-09-11

**Authors:** Jiayu Miao, Zhuxiao Ren, Yunbei Rao, Xin Xia, Jianlan Wang, Fang Xu, Xiaoling Zhang, Jie Yang

**Affiliations:** 1 Department of Neonatology, Guangdong Women and Children Hospital, Guangzhou, China; 2 Department of Neonatology, Guangzhou Medical University Second Affiliated Hospital, Guangzhou, China

**Keywords:** Chorioamnionitis, Amnionitis, Complications, Preterm infants, Intraventricular hemorrhage

## Abstract

**Objective:**

To investigate the effect of pathological staging of chorioamnionitis (CA) on complications in preterm infants;

**Methods:**

A single-center, retrospective study was conducted to choose singleton preterm infants (gestational age < 37 weeks) from the Department of Obstetrics and Gynecology in our hospital from December 2016 to December 2017. The basic data and placental pathological results were retrospectively collected. According to the placental pathological results of whether inflammation infiltrating amnion, CA 0/I phase was classified into non-amnionitis group, CA II/III phase was classified into amnionitis group, the incidence of common complications in preterm infants was compared. Further, logistic regression was used to analyze the effects of amnionitis on complications after being adjusted to gestational age, birth weight and thrombocytopenia.

**Results:**

A total of 221 preterm infants were enrolled, including 186 cases in non-amnionitis group and 35 cases in amnionitis group. The gestational age of amnionitis group (32.00 ± 2.71 weeks) was significantly lower than non-amnionitis group (34.14 ± 2.06 weeks), birth weight (1.93 ± 0.64 kg) was significantly lower than that of non-amnionitis group (2.26 ± 0.58 kg), and the hospital stay in amnionitis group was significantly longer (25.71 ± 19.23 days), all of the difference above was statistically significant(*P* < 0.05). The incidence of intraventricular hemorrhage (IVH) in amnionitis group (37.14%) was significantly higher than that in non-amnionitis group (13.98%) (*P =* 0.002), and the risk of IVH was significantly increased by amnionitis (OR = 3.636, 95%CI: 1.632–8.102); after correction of gestational age, birth weight and thrombocytopenia, the risk of IVH was still significantly increased (OR = 2.471, *P* = 0.046, 95% CI: 1.015–6.015). And the late-onset IVH was more common (*P* = 0.009).

**Conclusion:**

Amnionitis leads to a significant reduction in gestational age and birth weight in preterm infants, and it is an independent risk factor for IVH.

## Background

Chorioamnionitis, an inflammatory status of the intrauterine or fetal membrane, were divided into histological chorioamnionitis and clinical chorioamnionitis [1]. It is well known that chorioamnionitis can increase the incidence of intraventricular hemorrhage (IVH), periventricular leukomalacia (PVL), bronchopulmonary dysplasia (BPD), retinopathy (ROP), early-onset sepsis (EOS), late-onset sepsis (LOS), and necrotizing entercolitis (NEC) [2]. Chorioamnionitis leads to an inflammatory response in the placenta, inflammatory cells and factors could induce vascular endothelial injury leading to perfusion defect in placenta [3]. Placental malperfusion was associated with earlier preterm birth accompanied by impaired fetal growth and severe neonatal complications [4]. On the other hand, Chorioamnionitis can cause fetal inflammatory response syndrome (FIRS), which is a subclinical state caused by the activation of the fetal immune system and the release of a large number of inflammatory factors (IL-6, IL-1, IL-8, TNF-α). These inflammatory factors could interfere with the normal expression of fetal brain cytokines, leading to brain damage in preterm infants [5]. Some preclinical studies showed that fetal lung epithelial damage caused by chorioamnionitis could lead to impaired vascular osmotic pressure and apoptosis [6], which increased the secretion of lung surface proteins, but could also make the alveolar cell wall thinner [7, 8]. Similarly, inflammatory factors could damage the fetal lung capillary endothelial cells, resulting in damage to the alveoli, vascular structure and stagnation of alveolarization, which ultimately leads to the occurrence of BPD [9, 10]. Gantert. et al. confirmed that premature delivery and intrauterine inflammation affected the maturation of the fetal intestinal barrier, the intestinal tight junction protein-1 (TJP-1) damaged by bacterial toxins during gestation, resulting in an easy access of microbial toxins to the mucosa and the inner layers of the gut ante- and postnatally [11].

In summary, Chorioamnionitis is associated with adverse outcomes in preterm birth. Although, the effect of chorioamnionitis on complications in preterm infants has been widely studied, the pathological staging on them has been rarely published. Therefore, this study aimed to investigate the effect of pathological staging of chorioamnionitis on complications in preterm infants.

## Methods

### Study population

A single-center, retrospective study was conducted. Singleton infants (gestational age < 37 weeks) who were given birth in our hospital from December 2016 to December 2017 were selected as study population, cases of genetic metabolic diseases, nervous system malformations, and stillbirth were eliminated. The inclusion criteria for sending placenta for pathology investigation in our center are one of the following situations: 1. Mother with gestational diabetes or preeclampsia; 2. Mother had fever a week before or during delivery; 3. Macroscopic abnormity during delivery. In addition, informed consent for pathology analysis should be obtained. The protocol was approved by Ethics Committee of Guangdong Women and Children’s Hospital (201801053).

### Histopathological examination

After delivery, the whole placenta, fetal membrane and umbilical cord were fixed in 4% formaldehyde for 48 h, the quality of the placenta, the length and the attachment of umbilical cord, the number of blood vessels were evaluated, and the placenta was cut at intervals of 1 cm. All specimens were embedded in paraffin, sectioned, and routine Hematoxylin-Eosin Staining (HE staining).

The criteria for histological chorioamnionitis: Stage 0 (CA0): Normal placenta; Stage 1 (CAI): acute inflammation of the chorioamniotic membranes, in which neutrophilic infiltration is limited to the chorion; Stage 2 (CAII): acute inflammation of the chorioamniotic membranes, showing neutrophilic migration into the amniotic connective tissue (asterisk); Stage 3 (CAIII): acute inflammation of the chorioamniotic membranes, whose characteristic is the amnion epithelial necrosis (arrows) [12].

According to the placental pathological results of whether inflammation infiltrating amnion, CA 0/I phase was classified into non-amnionitis group, CA II/III phase was classified into amnionitis group.

### Clinical characteristics

The gestational age (gestational age in our data was based on date of last menstrual period with confirmation by ultrasound), Apgar score, birth weight, blood routine, C-reactive protein, procalcitonin and blood culture results of each premature infant were collected, and the disease of each system during hospitalization was observed. The relevant complications were as follows [13]:

Intraventricular hemorrhage (IVH), periventricular leukomalacia (PVL) are common complications in preterm infants, which can be detected by serial head ultrasound and MRI, the first is recommended between 5 and 8 days, 21 and 28 days, between 34 and 36 weeks of corrected gestational age [14]. Those with IVH born ≤3 d were defined as early-onset intraventricular hemorrhage, and > 3 d were defined as late-onset intraventricular hemorrhage [15].

The sepsis in this study requires positive clinical or laboratory screen and positive culture. Early-onset sepsis (EOS) occurred during the first 72 h of life, Late-onset sepsis (LOS) refers to sepsis that occurred between day of 4 and 120 [16].

Bronchopulmonary dysplasia (BPD) is defined as oxygen dependency at 36 weeks of corrected gestational age, the main causes including damage of alveolar, vascular structural and stagnation of alveolarization.

NEC is a syndrome manifested as abdominal distension, vomiting, diarrhea and Bloody stools, severe cases even develop shock and multiple systemic organ failure, can be diagnosed by abdominal piece, usually characterized by gas accumulation in the intestinal wall.

Retinopathy of prematurity (ROP) refers to retinal vasoconstriction, obstruction and retinal vascular hypoxia, leading to a large number of angiogenic factors, stimulating neovascularization form.

Neonatal thrombocytopenia: The platelet count ≤150*10^9^/L in all healthy newborn infants, regardless of gestational age [17].

### Statistical analysis

One Sample Kolmogorov—Smirnov test was performed for normality of distributions, and the normal distribution data was represented as the mean ± standard deviation (SD). Dichotomous data was expressed by the frequency and relevant percentage. Maternal and neonates’ characteristics were compared using χ^2^ and *t* tests as appropriate. Variables with statistical significance after univariate analysis and factors which could affect neonatal complications were further adjusted by multivariate logistic regression, the effect size is expressed in OR and 95% CI. Significance was accepted at *p* < 0.05. All analyses were performed by using SPSS version 19.0 software.

## Results

### General situation

A total of 221 maternal placenta were detected in this study. Including 186 cases in non-amnionitis group, 35 cases in amnionitis group. The gestational age of amnionitis group (32.00 ± 2.71 weeks) was significantly lower than non-amnionitis group (34.14 ± 2.06 weeks), birth weight (1.93 ± 0.64 kg) was significantly lower than that of non-amnionitis group (2.26 ± 0.58 kg), and the hospital stay in amnionitis group (25.71 ± 19.23 days) was significantly longer than that in non-amnionitis group (18.84 ± 16.80 days), the difference was statistically significant(*P* < 0.05). However, there were no significant difference in maternal age, placental weight, use of antenatal glucocorticoids, premature rupture of membranes, mode of delivery, gender, thrombocytopenia, and Apgar scores (*P* > 0.05). (As shown as in Table 1).

**Table 1:** The clinical characteristics of non- amnionitis group and amnionitis group

Clinical characteristics	Non-amnionitis group (186)	Amnionitis group (35)	*P*
Maternal age(y)	30.62 ± 5.38	30.89 ± 6.80	0.797
Gestational age(d)	34.14 ± 2.06	32.00 ± 2.71	<0.001
Placental weight(g)	480.71 ± 150.43	475.86 ± 106.68	0.856
Birth weight (kg)	2.26 ± 0.58	1.93 ± 0.64	0.003
Antenatal glucocorticoids,n,(%)	46(24.7%)	12(34.3%)	0.239
Premature rupture,n,(%)	64(34.0%)	16(45.7%)	0.186
Cesarean section, n,(%)	96(51.6%)	16(45.7%)	0.522
Male, n, (%)	107(57.5%)	16(45.7%)	0.197
Apgar score 1 min ≤ 7, n, (%)	7(3.8%)	3(8.6%)	0.417
Apgar score 5 min ≤ 7, n, (%)	4(2.2%)	1(2.9%)	1.000
PLT,≤150 × 10^9^/L, n, (%)	12(6.5%)	1(2.9%)	0.662
Hospitalization(d)	18.84 ± 16.80	25.71 ± 19.23	0.031

*PLT* Platelet. Data are expressed as n (%) or mean ± standard deviation. The measurement data were analyzed by independent sample t test, and the count data were analyzed by chi-square test. *P < 0.05, the difference was statistically significant

### Neonatal outcome

Among the 221 patients, the incidence of intraventricular hemorrhage (IVH) in amnionitis group (37.14%) was significantly higher than that in non-amnionitis group (13.98%), the difference was statistically significant (OR = 3.636, *P* = 0.002, 95%CI: 1.632–8.102); After correction of gestational age, birth weight and thrombocytopenia-the three factor that are regarded to affect the IVH incidence, the risk of IVH was still significantly increased (OR = 2.471, *P* = 0.046, 95% CI: 1.015–6.015), and it more likely to cause late-onset IVH (*P* = 0.009) (as shown as in Table 2); However, there was no difference between the two groups on the incidence of necrotizing enterocolitis (NEC), bronchopulmonary dysplasia (BPD), retinopathy of prematurity (ROP), sepsis (*P* > 0.05) (as shown as in Table 3). Therefore, it is indicated that histological amnionitis was an independent risk factor for intraventricular hemorrhage in premature infants, which is not affected by gestational age, birth weight and thrombocytopenia, and it had no significant effects on other common complications related to premature birth (*P* < 0.05).

**Table 2:** Comparison of chorioamnionitis staging and IVH staging

IVH staging	Non-amnionitis group (186)	Amnionitis group (35)	*P*
IVH, n, (%)	26(13.98%)	13(37.14%)	0.002
Early-onset, n, (%)	14(7.53%)	6(17.14%)	0.069
Late-onset, n, (%)	12(6.45%)	7(20.00%)	0.009

*IVH* Intraventricular hemorrhage. Data are expressed as n (%).The count data were analyzed by chi-square test. *P < 0.05, the difference was statistically significant

**Table 3:** Neonatal outcomes of non- amnionitis group and amnionitis group

Outcomes	Non- amnionitis group (186)	Amnionitis group (35)	*P*	*OR(95%CI)*	*Adjusted *P*	*Adjusted OR(95%CI)
IVH,n, (%)	26(13.98%)	13(37.14%)	0.002	3.636 (1.632–8.102)	0.046	2.471 (1.015–6.015)
BPD,n,(%)	7(3.8%)	3(8.6%)	0.222	2.397 (0.589–9.759)	0.444	0.493 (0.080–3.015)
NEC,n, (%)	20(10.8%)	3(8.6%)	0.699	0.778 (0.218–2.774)	0.117	0.322 (0.078–1.331)
ROP,n, (%)	16(8.6%)	2(5.7%)	0.569	0.644 (0.141–2.934)	0.104	0.251 (0.048–1.328)
Sepsis, n, (%)	1(0.5%)	1(2.9%)	0.235	5.441 (0.332–89.106)	0.415	3.740 (0.157–89.228)

*IVH* Intraventricular hemorrhage, *BPD* Bronchopulmonary dysplasia, *NEC* Necrotizing entercolitis, *ROP* Retinopathy. Data are expressed as n (%).The count data were analyzed by chi-square test. *P < 0.05, the difference was statistically significant.*Adjusted OR (adjusted for GA and BW, for IVH adjusted for GA and BW and thrombocytopenia)

## Discussion

### Principal findings

Intra-amniotic infection has been considered to be the cause of acute histologic chorioamnionitis and funisitis, and funisitis is the hallmarks for the FIRS, which is associated with the preterm labor, a higher rate of neonatal morbidity (after adjustment for gestational age), and multi-organ fetal involvement [12, 18]. In this prospective study performed on 221 preterm newborns, we assessed their birth outcomes and common complications following amnionitis exposure. The key findings of this study are the following: (1) Placental amnionitis could lead to intrauterine growth retardation, associated with lower birth weight and preterm birth. (2) Preterms in amnionitis group had a higher occurrence of IVH, after correction of the common risk factors in IVH, including gestational age, birth weight and thrombocytopenia, amnionitis is still an independent risk factor for IVH.

### Strengths and weaknesses

The study has three strengths. First, we divided the studying population into non-amnionitis and amnionitis groups, the classification was not reported previously and it could highlight the effect of severe intrauterine inflammation on birth outcomes and complications of preterm compared with other previous studies. The placental tissue inflammation continues to progress and the intensity of the inflammation increases, which can cause severe amniotic inflammation and fetal inflammatory response syndrome, it is believed that the latter is closely related to the occurrence of premature delivery and fetal complications [19, 20]. Second, gestational age was confirmed by ultrasound, which is more rigorous. Third, histological chorioamnionitis may still be present in case of premature labor, even in the absence of clinical signs and premature rupture of membranes. Therefore, we use histological criteria for amnionitis. However, this study also has some limitations. First, the number of amniotitis cases was limited and no placental pathology was done in stillbirth. Second, except for postpartum factors, some prenatal factors may also cause IVH, such as placental vascular disease, hemodynamics, which our study did not evaluate. Third, Gomez et al. found that the content of IL-6 in fetal umbilical cord blood was an independent risk factor for severe complications in FIRS, IL-6 > 11 pg / mL as FIRS reference standard [21], but in our study, we didn’t give the evidence between CA and FIRS. Therefore, we may conduct a larger study to assess the effect of pathological staging of CA on preterm infants.

### Comparison with previous studies

The placenta functions are important for development of fetal multiple organs and systems. Placental dysfunction would affect the fetus, resulting in fetal growth restriction, premature and neurodevelopmental abnormalities [22, 23]. A series of studies have incorporated placental features into studies of stillbirth, preterm delivery and growth restriction [24–27]. Erdemir et al. revealed that histological chorioamnionitis led to earlier gestational age and lower birth weight [28]. Zanardo V et al. reported that the incidence of IVH in premature infants born to pregnant women with CA could reach 16.1%, and the stage 3 of placental inflammation could increase the risk of IVH by 3.2 times [29]. This study found that the CAII/III is a risk factor for IVH in premature infants, which is consistent with reports in the literature. However, for the staging of IVH, many studies have not specifically emphasized the role of chorioamnionitis [30–32], IVH of early-onset is known to be associated with greater neonatal mortality rate and the risk of subsequent neurodevelopmental disability in survivors [30, 33]. Therefore, it is important to understand whether CA plays a role in the pathogenesis of early-onset IVH as opposed to late-onset IVH. Subrata et al. showed that chorioamnionitis was not associated with increased risk of early IVH [15]; Lou reported that the higher incidence of IVH associated with CA might have been mostly of late onset [34]. In our study, the late-onset IVH was more predominant(*P*<0.05), which was the same with Lou. Chorioamnionitis leads to the release of vasoactive inflammatory cytokines which can mediate blood–brain barrier alterations, intra-vascular cells adhesion, coagulation, and thrombosis, conversely, this can lead to endothelial damage. If the severity of systemic and local hemodynamic changes increased in infants accompanied by cerebral endothelial damage, the enhanced late neonatal inflammatory response syndrome leads to the development of late onset IVH [15].

Earlier studies have focused mainly on the respiratory and neurological outcomes, chorioamnionitis have more recently been described in several other organs and systems. Watterberg et al. were the first to show that exposure to placental inflammation decreased the risk of RDS, but a subsequent increase in chronic lung injury (BPD) [35]. But a few studies have reported no relationship between placental inflammation and RDS [36], but our research lacks of the investigation on RDS. Some studies indicate that histologic chorioamnionitis could increase the risk of early onset sepsis and reduce the risk of late onset sepsis in preterm infants [37, 38], However, we have not been able to explore the early-onset and late-onset in our study. In addition, there were studies reported that chorioamnionitis seemed to contribute importantly to the development of ROP and NEC [39, 40], which our study still has not been able to get same findings.

Antenatal factors are also important to chorioamnionitis and neonatal morbidity. Antenatal steroids were reported to reduce negative effects of CA, such as IVH and RDS, as well as mortality by suppression of inflammation [41]. In this study, antenatal steroids did not change the frequency of CA in pregnant women, but it could be evaluated for neonatal morbidities by multiple regression analysis. Erdemir and his colleague analyzed the effect of many maternal risk factors on CA, neonatal morbidity and mortality rates, not only common complications, but also the severity of illness such as ventilation duration, surfactant therapy [28], which is lacking in our study.

### Meaning of the study

This study revealed the adverse birth outcomes caused by severe intrauterine inflammation exposure, which could help to highlight the importance of placental pathology examinations, therefore guiding clinical management and reducing the devastating burden of adverse birth outcomes.

### Unanswered questions and future research

In this study, there is a lack of the underlying mechanisms of stage of chorioamnionitis on neonatal adverse outcomes. For example, how the chemotactic signals in the amniotic cavity induced the amniotic inflammation, the relationship of FIRS and IVH. Therefore, additional preclinical studies are needed to explore these problems, and the number of included amnionitis cases needs to be enlarged.

## Conclusions

Histological chorioamnionitis is closed to various complications of premature infants, especially intraventricular hemorrhage IVH associated with a fetal inflammatory response syndrome [42]. Clinically, the effect of intrauterine inflammation on premature infants should be emphasized. For mothers with chorioamnionitis, focus on monitoring, especially that the placenta should be routine pathological examination, improve the detection rate of chorioamnionitis, reduce the sequelae of nervous system in premature infants.

## Data Availability

Please contact author for data requests.
